# The Transactions of the World’s Columbian Dental Congress

**Published:** 1894-03

**Authors:** 


### The Transactions of the World's Columbian Dental Congress.

CHICAGO, FEB. 22, 1894.

The Transactions are now nearly ready for the final proof-reading, it is therefore very desirable that those who want extra copies, and those who would like to subscribe for them do so at once, as it is the intention of the Committee of Publication to print only a sufficient number of copies to supply the members of the Congress and those who have subscribed for them. The subscription price is $10.00.

Subscriptions should be sent to Dr. John S. Marshall, Treas., 1003 Venetian Building, Chicago, Ill.

